# A Case of Diffuse and Nodular Glomerulosclerosis in Waldenstrom’s Macroglobulinemia

**DOI:** 10.7759/cureus.10694

**Published:** 2020-09-28

**Authors:** Dae Hyun Lee, Abhishek Kumar, Nicoleta Radoianu, Lindsey Ehren, Claude Bassil

**Affiliations:** 1 Cardiovascular Medicine, University of South Florida (USF) Health Morsani College of Medicine, Tampa, USA; 2 Nephrology, University of South Florida (USF) Health Morsani College of Medicine, Tampa, USA; 3 Internal Medicine, University of South Florida (USF) Health Morsani College of Medicine, Tampa, USA; 4 Nephrology, Moffitt Cancer Center, Tampa, USA

**Keywords:** nephrology, renal, waldenstrom, macroglobulinemia

## Abstract

Diffuse and nodular glomerulosclerosis is associated with diabetic nephropathy and occasionally with tobacco users. However, it has also been linked with amyloidosis, cryoglobulinemia, and light-chain deposition disease. To the best of our knowledge, there is no published data on diffuse and nodular glomerulosclerosis without light chain deposition in Waldenstrom’s macroglobulinemia (WM). We present a case of diffuse and nodular glomerulosclerosis in a non-diabetic, non-smoker with WM.

## Introduction

Waldenstrom’s macroglobulinemia (WM) is a type of non-Hodgkin’s lymphoma characterized by the accumulation of plasma cells in the bone marrow that characteristically secrete immunoglobulin M (IgM) protein [1]. It represents 2% of all hematological malignancies each year, with about 1000-1500 cases annually [2]. Generally speaking, kidney involvement occurs less often than in patients with multiple myeloma because of less light chain production. Despite IgM’s ability to aggregate in the subendothelium of the glomerular capillaries, the accumulation of monoclonal light chains as amyloid light-chain amyloidosis deposition leading to diffuse or nodular glomerulosclerosis is not common in patients with WM [3,4]. However, we report a case of a non-diabetic, non-smoking patient with the absence of any risk factors, developing diffuse and nodular glomerulosclerosis with WM.

## Case presentation

**Figure 1:**
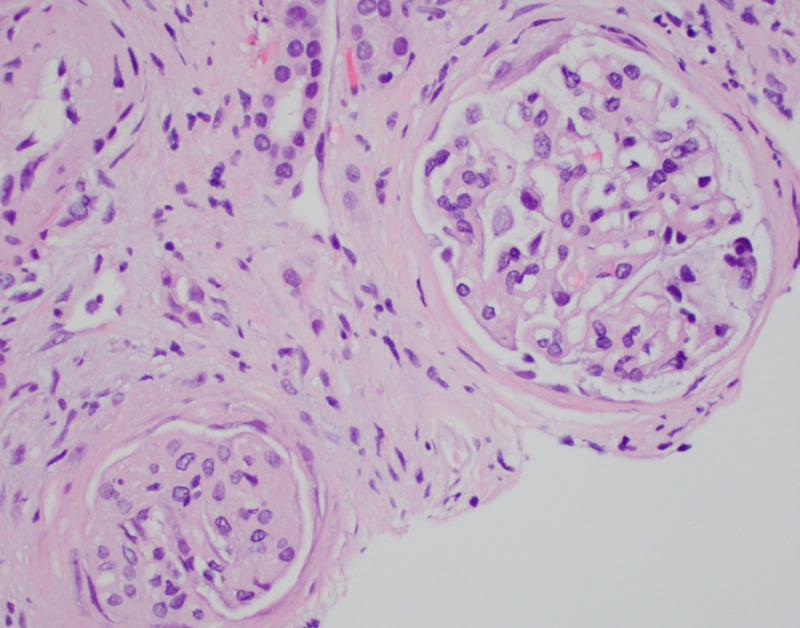
Kidney biopsy staining shows moderate diffuse and nodular glomerulosclerosis with focal cortical atrophy, hypoperfusion, and focal global and segmental glomerulosclerosis and moderate vascular sclerosis

A 72-year-old male with a past medical history of WM develops worsening renal function after cyclophosphamide/ prednisone plus rituximab (CP-R) treatment with partial remission. The patient does not have a history of diabetes, hypertension, or other comorbidities; he is a non-smoker. He used minimal nonsteroidal anti-inflammatory drugs (NSAIDs) and no recent or prior intravenous contrast use. HIV, syphilis, and hepatitis screens were unremarkable as possible contributors to worsening renal disease. Two months after completion of the CP-R regimen, creatinine started rising from baseline 1.0 mg/dL to 2.2 mg/dL over two years. Serum IgM decreased from 3414 to 2360 after CP-R treatment. Blood glucose, liver function panel, and albumin levels were normal. Renal ultrasound shows normal echogenicity without hydronephrosis. Kidney biopsy showed moderate diffuse and nodular glomerulosclerosis with focal cortical atrophy, hypoperfusion, and focal global and segmental glomerulosclerosis and moderate vascular sclerosis (Figure 1). Biopsy showed stronger background staining for kappa light chains compared to lambda light chains, without Randall-type electron-dense deposits. There was no evidence of monoclonal immunoglobulin deposition, amyloidosis, light-chain tubulopathy, or light-chain cast nephropathy. After three rounds of chemotherapy, the patient’s IgM levels went to 2128. Serum kappa levels continued on a downward trend during the patient’s treatment interval but have risen from the 1600s to 3400 most recently. The patient is currently in partial remission for his WM and is being monitored every three to six months. 

## Discussion

We present a case of a patient with WM who presented with worsening kidney damage subsequently diagnosed with diffuse and nodular glomerulosclerosis (DNGS). There are multiple risk factors known for the development of DNGS such as diabetes, IgA nephropathy, Henoch-Shönlein purpura, membranous nephropathy, and membranoproliferative glomerulonephritis [5]. The differential diagnoses for nodular glomerulosclerosis include renal amyloidosis, monoclonal immunoglobulin deposition disease, cryoglobulinemia glomerulosclerosis, fibrillar glomerulonephritis, immunotactoid glomerulonephritis, and idiopathic nodular glomerulosclerosis [5]. Diffuse and nodular glomerulosclerosis are usually asymptomatic until 5-15 years after onset [6]. Afterward, patients present with microalbuminuria and about 60% of them begin developing early stages of hypertension [6]. In a more advanced stage, they develop overt nephropathy, deterioration of GFR, and formation of Kimmelstiel-Wilson nodules [6]. Diagnosis and treatment should begin in stage 2 chronic kidney disease to prevent further progression of the disease. Patients need to be assessed for the previous history of hypertension or cardiovascular disease. In addition to risk factor assessment, other causes of DNGS need to be excluded as mentioned previously.

A comparison in the development of diffuse and nodular glomerulosclerosis in a patient with WM without any risk factors for development has not been well documented. There is one case describing deposition of light chain in the glomerulus in WM, but also with diabetes mellitus, a known risk factor development of glomerulosclerosis [7]. Additionally, the patient in the report had a worse renal failure than our patient, further exacerbating the condition. A study done comparing the incidence, pathology, and clinical outcomes of renal disease in 44 patients with WM showed that AL amyloidosis as the number one etiology of renal disease (n = 11, 25%), IgM deposition disease as second (n = 10, 23%), and lymphoplasmacytic lymphoma infiltration as third (n = 8, 18%). These all led to a decreased eGFR from baseline as well as the presence of nephrotic syndrome (8.1 g protein/24h) [8]. The principle similarities between nodular glomerulosclerosis and WM renal disease were the presence of light chain deposition disease. These two etiologies could be considered when the evidence of diffuse and nodular glomerulosclerosis is seen histologically and other contributors have been ruled out. On histology, the appearance of the glomerulus is non-specific, and immunofluorescent or electron micrographic studies would need to be performed to elicit the etiology [8]. Since our patient had diffuse and nodular glomerulosclerosis in addition to focal and segmental glomerulosclerosis, other etiologies needed to be excluded such as sickle cell disease, HIV, and IV drug abuse, all of which are associated with the development of focal segmental glomerulosclerosis (FSGS).

The current consensus on the treatment of DNGS is to prevent further progression of the disease by lower glomerular pressures using angiotensin-converting enzyme inhibitors (ACEI) or angiotensin receptor blocker (ARB) [7]. ACEIs or ARBs have not been used to treat primary FSGS specifically, but they have been shown to reduce the progressive proteinuria that accompanies FSGS similar to DNGS [9]. In our patient who had symptomatic renal disease, exploring these possibilities could be a possible treatment avenue. Treatment of WM with chemotherapy is generally considered to improve or stabilize WM patients with renal complications, but this may not be the optimal approach and further treatment options need to be explored [8].

The prognosis of DNGS is generally dependent upon four main risk factors: the degree of proteinuria, severity of renal dysfunction, histologic findings, and response of proteinuria to therapy [9]. Those with nephrotic syndrome with greater than 3.5 g/dL/day, but less than 10 g/dL/day had five-year survival rates between 60% and 90%. If proteinuria was over 10 g/dL/day, most patients eventually progressed to end-stage renal disease. Increased renal dysfunction is associated with decreased outcomes, specifically having creatinine concentrations > 1.3 mg/dL being associated with significantly decreased 10-year survival rates. The presence of interstitial fibrosis is associated with poor renal survival [10]. However, the most important determinant of renal outcomes was the initial response of proteinuria to therapy, specifically complete remission of proteinuria (defined as <200 to 300 mg/dL/day) being associated with much better renal outcomes. In our patient, he did not have any abnormal albumin levels as well as the absence of amyloidosis or other predisposing risk factors [10]. This further supports the rationale for identifying this unique case: a stable patient with the absence of clinically significant risk factors developed glomerulosclerosis. Therefore, it is worth noting that a small number of patients with WM can develop diffuse and nodular glomerulosclerosis despite the absence of classic risk factors [10].

## Conclusions

Here, we presented a case of diffuse and nodular glomerulosclerosis in a patient with WM. We entertained a variety of possible risk factors for the development of DNGS, all of which were absent from our patient. Different avenues for treatment to slow the progression of proteinuria associated with DNGS were also explored, but the presentation - a previously stable patient with the absence of clinically significant risk factors developing glomerulosclerosis - warranted a more personalized approach to his care. In order to determine the cause of his glomerulosclerosis, further investigation and exclusion of other possible etiologies that may not have been explored should be undertaken. The appearance of this patient will necessitate exploration into possible treatment options for these atypically presenting patients.
